# Correlations between Resting Electrocardiogram Findings and Disease Profiles: Insights from the Qatar Biobank Cohort

**DOI:** 10.3390/jcm13010276

**Published:** 2024-01-03

**Authors:** Fatima Qafoud, Khalid Kunji, Mohamed Elshrif, Asma Althani, Amar Salam, Jassim Al Suwaidi, Dawood Darbar, Nidal Asaad, Mohamad Saad

**Affiliations:** 1 College of Health Sciences, Qatar University, Doha P.O. Box 2713, Qatar; fkafood@hotmail.com (F.Q.); aaja@qu.edu.qa (A.A.); 2 Qatar Computing Research Institute, Hamad Bin Khalifa University, Doha P.O. Box 5825, Qatar; kkunji@hbku.edu.qa (K.K.); melshrif@hbku.edu.qa (M.E.); 3 Department of Cardiology, Al-Khor Hospital, Hamad Medical Corporation, Doha P.O. Box 3050, Qatar; asalam@hamad.qa; 4 Heart Hospital, Hamad Medical Corporation, Doha P.O. Box 3050, Qatar; jalsuwaidi@hamad.qa; 5 Division of Cardiology, Department of Medicine, University of Illinois Chicago, Chicago, IL 60612, USA; darbar@uic.edu

**Keywords:** ECG, arrythmia, risk scores, Qatar Biobank, type 2 diabetes, cardiovascular diseases, Middle East, diverse populations

## Abstract

Background: Resting electrocardiogram (ECG) is a valuable non-invasive diagnostic tool used in clinical medicine to assess the electrical activity of the heart while the patient is resting. Abnormalities in ECG may be associated with clinical biomarkers and can predict early stages of diseases. In this study, we evaluated the association between ECG traits, clinical biomarkers, and diseases and developed risk scores to predict the risk of developing coronary artery disease (CAD) in the Qatar Biobank. Methods: This study used 12-lead ECG data from 13,827 participants. The ECG traits used for association analysis were RR, PR, QRS, QTc, PW, and JT. Association analysis using regression models was conducted between ECG variables and serum electrolytes, sugars, lipids, blood pressure (BP), blood and inflammatory biomarkers, and diseases (e.g., type 2 diabetes, CAD, and stroke). ECG-based and clinical risk scores were developed, and their performance was assessed to predict CAD. Classical regression and machine-learning models were used for risk score development. Results: Significant associations were observed with ECG traits. RR showed the largest number of associations: e.g., positive associations with bicarbonate, chloride, HDL-C, and monocytes, and negative associations with glucose, insulin, neutrophil, calcium, and risk of T2D. QRS was positively associated with phosphorus, bicarbonate, and risk of CAD. Elevated QTc was observed in CAD patients, whereas decreased QTc was correlated with decreased levels of calcium and potassium. Risk scores developed using regression models were outperformed by machine-learning models. The area under the receiver operating curve reached 0.84 using a machine-learning model that contains ECG traits, sugars, lipids, serum electrolytes, and cardiovascular disease risk factors. The odds ratio for the top decile of CAD risk score compared to the remaining deciles was 13.99. Conclusions: ECG abnormalities were associated with serum electrolytes, sugars, lipids, and blood and inflammatory biomarkers. These abnormalities were also observed in T2D and CAD patients. Risk scores showed great predictive performance in predicting CAD.

## 1. Introduction

Resting electrocardiogram (ECG) is a valuable diagnostic tool used in clinical medicine to assess the electrical activity of the heart while the patient is at rest [1,2,3]. ECG abnormalities directly indicate certain diseases, such as atrial fibrillation (AF) [4] and heart arrhythmias (ARs) [5]. They tend to occur more often in patients with certain diseases (e.g., diabetes [6,7], coronary artery disease (CAD) [8], hypertension (HTN) [9]) compared to healthy people.

ECG abnormalities have been shown to be associated with metabolic syndrome and its components, with evidence changing by gender [10]. They have also been associated with insulin-induced hypoglycemia [11,12] and abnormal serum electrolyte levels. Deviations in the concentrations of extracellular potassium, calcium, and magnesium have the potential to disrupt the myocyte membrane potential gradients and modify the cardiac action potential [13]. Many of these results were obtained in disease cohorts and not in general populations.

Risk scores using ECG parameters have been proposed to predict various outcomes, including mortality [14], sudden cardiac death in the general population [15], and cardiovascular disease and its subclinical phenotypes [16,17,18]. These risk scores can be combined with traditional clinical risk scores that are used to predict cardiovascular diseases (CVD) (e.g., QRISK3) [19] and type 2 diabetes (T2D) (e.g., FINDRISC) [20]. Accurate disease prediction and early detection are important for better prevention and management of CAD and T2D.

Most findings about ECG association with various diseases and performance of risk scores were determined using cohorts of individuals of European descent. To the best of our knowledge, ECG was never studied in the Middle Eastern and North African (MENA) region. In this study, we evaluated the association between ECG traits and (1) diseases such as T2D and CAD, (2) serum electrolytes, (3) blood and inflammatory biomarkers (e.g., red blood cells, white blood cells, C-reactive protein, etc.), and (4) CVD/T2D risk factors (e.g., LDL-C, insulin, etc.) in the Qatar Biobank (QBB) dataset. We used the 12-lead resting ECG data of 13,827 subjects from QBB, a self-reported questionnaire, biochemical markers (serum electrolytes and CVD/T2D risk factors (sugars/lipids/BP)), and available electronic medical records (EMRs) of 8308 subjects to extract disease information (e.g., CAD, AF, etc.).

## 2. Materials and Methods

### 2.1. Study Cohort

The dataset consisted of 13,827 Qatari individuals. QBB collected all samples and generated the phenotypic data [21]. Personal referrals from family, friends, social media, and the QBB’s website were used to recruit participants. Deep phenotyping was performed at QBB facilities. Participants filled out a standardized questionnaire presenting information about lifestyle, nutrition, and medical history. Blood, saliva, and urine were collected and kept in liquid nitrogen at –80 °C. To ensure informed permission from all participants, the research study protocol’s ethical approval was obtained from the Hamad Medical Corporation Ethics Committee (Protocol No. MRC-03-20-097) and QBB Institutional Review Board (IRB) (Protocol No. E-2019-QF-QBB_RES-ACC-0153-0103) in 2020 and renewed on an annual basis.

### 2.2. Phenotypic Data: Serum Electrolytes, Sugars/Lipids, Blood and Inflammatory, and Clinical/Disease Traits

EMRs for 8308 participants were available based on the International Classification of Diseases, 10th Revision Codes and Systemized Nomenclature of Medicine Clinical Terminology Codes (SNOMED CT). Self-reported questionnaires and biochemical marker data were available on 14,259 subjects. Five categories were considered: (1) demographics, which included sex, age, BMI, and ancestry; (2) serum electrolytes, which included chloride, magnesium, potassium, sodium, calcium, phosphorus, and bicarbonate; (3) sugars/lipids, which included glucose, HbA1C, insulin, LDL-C, HDL-C, total cholesterol (TC), triglycerides (TG); (4) blood and inflammatory, which included eosinophil, basophil, lymphocyte, monocyte, neutrophil, red blood cells, white blood cells, and C-reactive protein; and (5) clinical/disease traits, which included CAD (527 patients), T2D (3308 patients), AF (67 patients), AR (391 patients), cardiomyopathy (CM; 48 patients), stroke (92 patients), hyperthyroidism (1369 patients), smoking (3615 patients), systolic blood pressure (SBP), and diastolic blood pressure (DBP). T2D status was determined using HbA1C (T2D if HbA1C > 6.5), self-reported questionnaire, and available EMR data. CAD, AF, CM, stroke, hyperthyroidism, and smoking were extracted from EMR and questionnaire data. Note that all ECG traits and tested continuous variables were mean-centered and standardized.

### 2.3. ECG Data

Resting 12-lead ECG was performed using Mortara Eli 350 or 380 automated system (Welch Allyn, Skaneateles Falls, New York, NY, USA) to collect data from participants who were required to rest for 2 min. The ECG data were collected following 10 s ECG recording with an interval of 1 min between each recording, three times [21]. Measures were automatically recorded, including the RR interval, PR interval, QRS duration, and corrected QT interval (QTc). These measures were averaged over the 3 recordings and used for analysis. Two additional variables were calculated: P wave (PW) was calculated as max (Offset–Onset) over the three time points, and JT interval as “average QTc–average QRS”. ECG data were available for 13,827 participants.

### 2.4. Statistical Analysis

Association analysis was performed between the 6 ECG traits (RR, PR, QRS, QTc, PW, and JT) and each of the demographics, serum electrolytes, sugars/lipids, blood and inflammatory, and clinical/disease traits. Linear regression was applied, adjusting for sex, age, and BMI, except when testing the association with demographic variables. Risk scores were developed to predict CAD using a variety of models that included the following as predictors: demographics only, ECG traits only, serum electrolytes only, sugars/lipids only, and clinical/disease traits that are known to be risk factors for CAD. A global model was developed, including all traits from the previous categories. The risk scores were created by splitting the full data into training and testing datasets (70% vs. 30%, respectively). Multivariate logistic regression and xgboost machine-learning models were performed. In the multivariate regression model (RS_mult_), the regression effect sizes were recorded and used to build the risk score as a weighted sum (effect size × predictors). Only predictors that had a *p* < 0.05 were included in the risk score calculation. Xgboost risk score (RS_xgboost_) included all features/predictors and used the following parameters: max_depth = 2, gamma = 0, max_delta_step = 0, lambda = 1, eta = 0.001, nthread = 8, and nrounds = 4000 in the xgboost R package (https://cran.r-project.org/web/packages/xgboost/index.html, accessed on 1 September 2023). The performance of each score was evaluated using logistic regression between the disease and the risk score. OR per 1 SD increase, the area under the receiver operating curve (AUC), and OR for the top decile (OR_decile_) vs. the remaining deciles were reported as performance metrics in the testing dataset.

## 3. Results

The cohort characteristics and summary of the ECG traits before normalization are shown in Table 1. The total number of subjects was 13,827, of which 44.46% were male. The cohort was relatively young, with an average age of 40.12 ± 13.11. The average BMI was 29.6 ± 6.16.

### 3.1. Association Analysis

#### 3.1.1. Demographics

**Figure 1:**
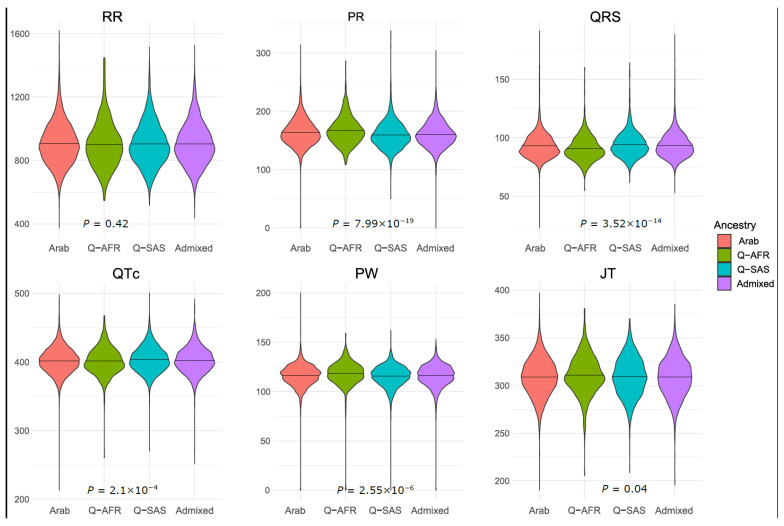
Variability in ECG traits by ancestry. Q-AFR: Qatari citizens with African origins; Q-SAS: Qatari citizens with South Asian origins (Iran and India); Arab: Qatari citizens with Arab origins spanning the Gulf and Middle East region.

Sex, age, and BMI were all associated with all ECG traits (*p* < 3.8 × 10^−13^; Table 2). RR, PR, QRS, and PW were significantly elevated in females, with QRS being the most significant ECG trait. QTc and JT were significantly decreased in females (Table 2). All ECG traits except for RR were positively correlated with age and BMI (Table 2). Age showed the most significant association with QTc (*p* = 3.4 × 10^−218^) and BMI with PW (*p* = 9.1 × 10^−222^) (Table 2). Ancestry, as inferred using genetic data, was associated with ECG traits. Only JT and RR did not show significant associations using the Bonferroni significance level (α = 0.05/6 = 0.008) (Figure 1). PR was the ECG trait with the largest differences between ancestral groups (*p* = 7.99 × 10^−19^; Figure 1). PR was the largest in individuals with African origins (average—167 ms) and lowest in individuals with South Asian origins (average—159 ms). QRS was the largest in South-Asian-origin individuals (average—94 ms) and the lowest in African-origin individuals (average—91 ms) (Figure 1). QTc was also the highest in South-Asian-origin individuals. Although differences were observed between ancestral groups, the magnitude of these differences may not be of clinical relevance.

#### 3.1.2. Serum Electrolytes

**Figure 2:**
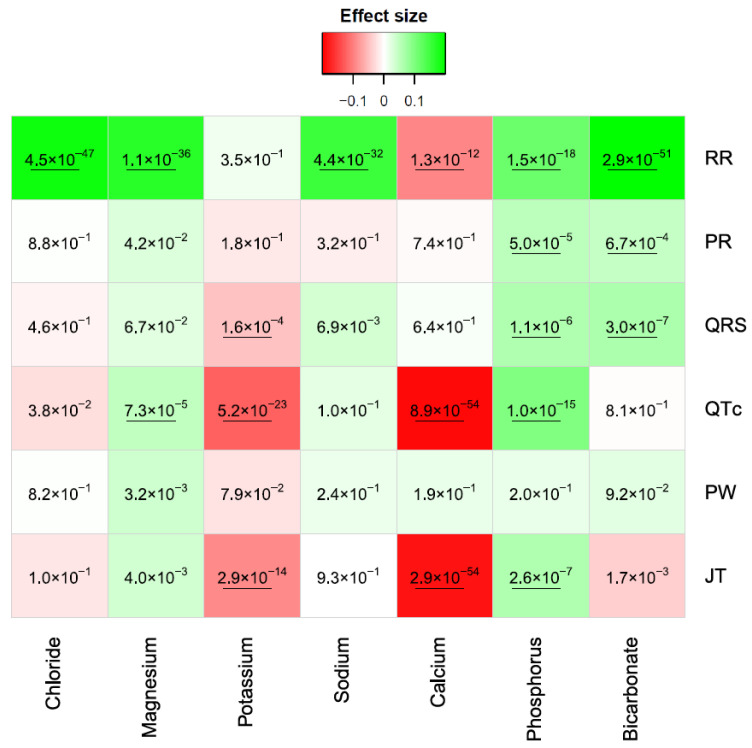
Associations between ECG traits and serum electrolytes. Colors are proportional to the effect size of each regression model. Red colors represent a negative correlation, while green colors represent positive ones. The numbers in each cell are the *p*-values. The underlined *p*-values are significant with Bonferroni threshold.

With Bonferroni significance (0.05/(7 × 6) = 1.1 × 10^−3^), RR was significantly associated with all tested electrolytes except potassium (Figure 2). Electrolytes were associated with an increase in RR, except for calcium (Figure 2). PR was only associated with phosphorus and bicarbonate, showing a positive correlation (Figure 2). QRS was associated with potassium (negative correlation) and phosphorus and bicarbonate (positive correlations) (Figure 2). QTc was significantly decreased with potassium and calcium but increased with phosphorus and magnesium (Figure 2). PW did not show significant associations at Bonferroni levels, while JT was significantly elevated with phosphorus and decreased with potassium and calcium (Figure 2).

#### 3.1.3. Sugars/Lipids

**Figure 3:**
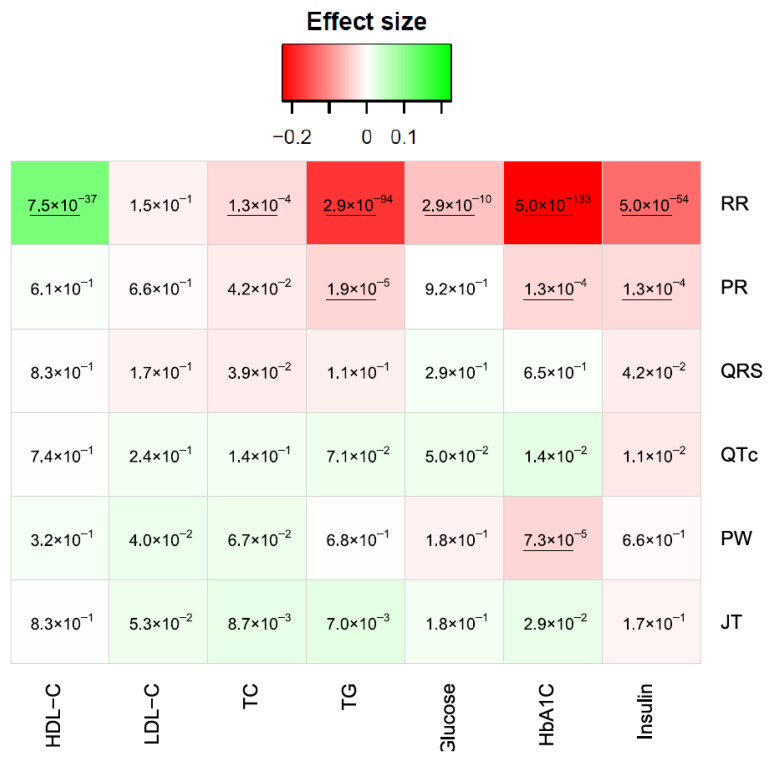
Associations between ECG traits and sugars/lipids. Colors are proportional to the effect size of each regression model. Red colors represent a negative correlation, while green colors represent positive ones. The numbers in each cell are the *p*-values. HDL-C: high-density lipoprotein cholesterol; LDL-C: low-density lipoprotein cholesterol; TC: total cholesterol; TG: triglyceride. The underlined *p*-values are significant with Bonferroni threshold.

With Bonferroni significance (0.05/(7 × 6) = 1.1 × 10^−3^), all sugars/lipids traits except LDL-C were associated with RR (Figure 3). Only HDL-C showed a positive correlation with RR, meaning that the increase in HDL-C was associated with an increase in RR (Figure 3). The two most significant associations were between RR and HbA1C (*p* = 5.0 × 10^−133^) and between RR and TG (*p* = 2.9 × 10^−94^) (Figure 3). For PR, only insulin, HbA1C, and TG showed significant associations (*p* = 1.3 × 10^−4^, 1.3 × 10^−4^, and 1.9 × 10^−5^, respectively; Figure 3). For QRS, QTc, PW, and JT, only one negative association was significant between PW and HbA1C (*p* = 7.3 × 10^−5^) (Figure 3).

#### 3.1.4. Blood and Inflammatory

**Figure 4:**
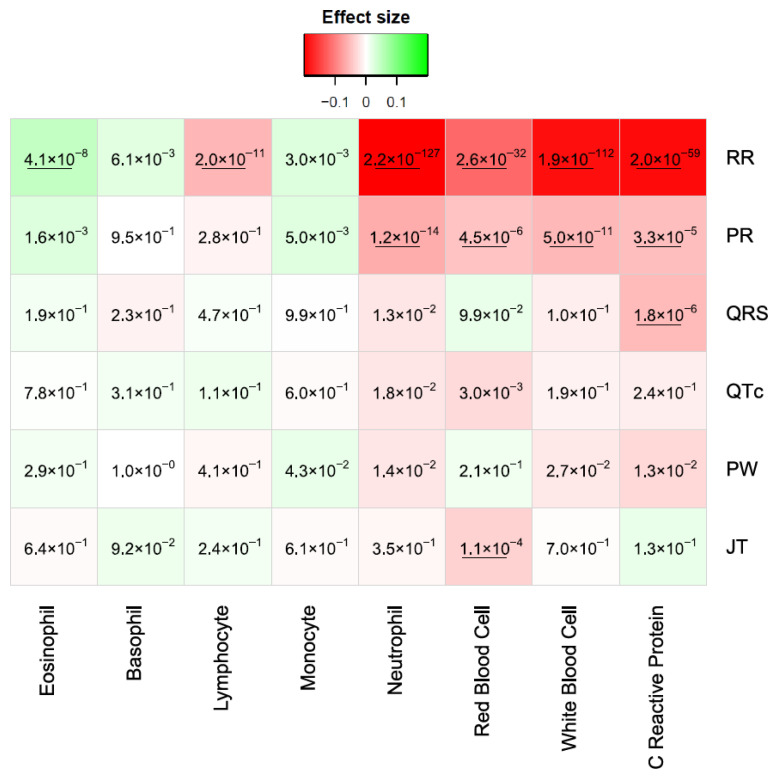
Associations between ECG traits and blood and inflammatory biomarkers. Colors are proportional to the effect size of each regression model. Red colors represent a negative correlation, while green colors represent positive ones. The numbers in each cell are the *p*-values. The underlined *p*-values are significant with Bonferroni threshold.

Here, the Bonferroni significance level was 0.05/(8 × 6) = 10^−3^. Monocytes and basophils did not show significant associations with RR (Figure 4). Eosinophils were associated with an elevated RR, while the remaining blood traits were associated with a decreased RR (Figure 4). For PR, neutrophils, red blood cells, white blood cells, and C-reactive protein were significantly associated and negatively correlated with PR (*p* = 1.2 × 10^−14^, 4.5 × 10^−6^, 5.0 × 10^−11^, and 3.3 × 10^−5^, respectively; Figure 4). For QRS and JT, C-reactive protein and red blood cells yielded negative associations (*p* = 1.8 × 10^−6^ and 1.1 × 10^−4^, respectively; Figure 4). No significant associations were observed for QTc and PW (Figure 4).

#### 3.1.5. Clinical/Disease Traits

**Figure 5:**
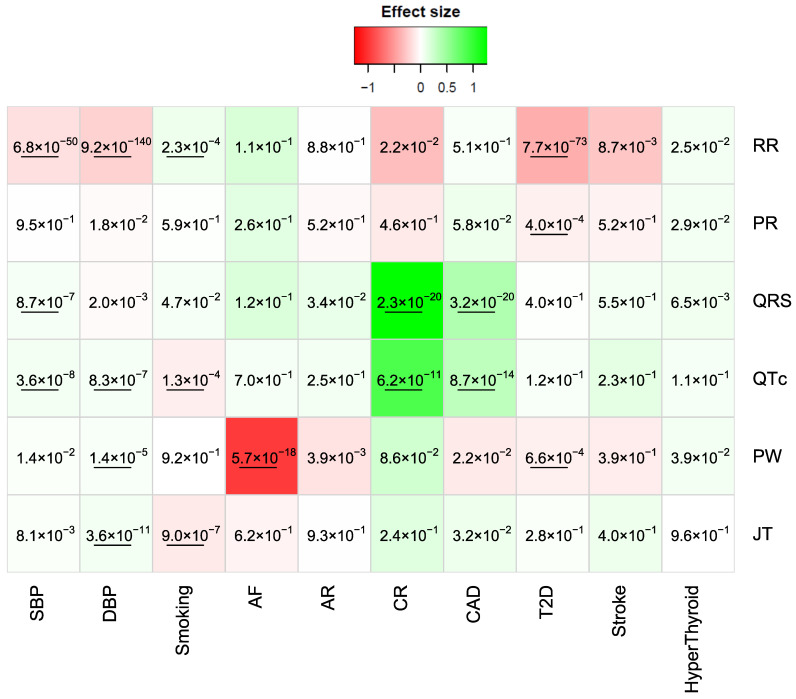
Associations between ECG traits and clinical/disease traits. Colors are proportional to the effect size of each regression model. Red colors represent a negative association, while green colors represent positive ones. The numbers in each cell are the *p*-values. Cases were coded as 1 and controls as 0. AF: atrial fibrillation; AR: arrhythmia; CAD: coronary artery disease; T2D: type 2 diabetes; HyperThyroid: hyperthyroidism. The underlined *p*-values are significant with Bonferroni threshold.

AF was significantly associated with PW at a Bonferroni significance level (α = 0.05/(10 × 6) = 8.3 × 10^−4^; *p* = 5.7 × 10^−18^; Figure 5). Low PW was associated with a higher risk of AF (Figure 5). Higher CM risk was associated with elevated QRS and QTc (*p* = 2.3 × 10^−20^ and 6.2 × 10^−11^, respectively; Figure 5). Increased QRS and QTc were associated with elevated risk of CAD (*p* = 2.3 × 10^−20^ and 6.2 × 10^−11^, respectively; Figure 5). T2D risk was decreased with elevated PW (*p* = 6.6 × 10^−4^), elevated PR (*p* = 4 × 10^−4^), and elevated RR (*p* = 7.7 × 10^−73^) (Figure 5). Hyperthyroidism and stroke were not associated with any ECG traits (Figure 5). SBP and DBP were associated with RR (*p* = 6.8 × 10^−50^ and 9.2 × 10^−140^, respectively) and QTc (*p* = 3.6 × 10^−8^ and 8.3 × 10^−7^, respectively). QRS was associated with SBP (*p* = 8.7 × 10^−7^), JT was associated with DBP (*p =* 3.6 × 10^−11^), and PW was associated with DBP (*p =* 1.4 × 10^−5^) (Figure 5).

### 3.2. Risk Score Performance to Predict CAD

Risk score results are presented in Table 3. The RS_mult_ for the demographic variables was performed twice. In the first model, only sex and age were the variables that contributed to the risk scores, while BMI and ancestry were not significant. The OR was 3.84 (95% CI [3.21, 4.59], *p* = 3.55 × 10^−49^) and AUC was 0.84. However, this performance is due to the data collection process and the unbalanced distribution of age and sex for CAD vs. control subjects (mean age in CAD = 54.46 vs. 39.57 in controls; 39% of CAD patients were females, whereas 56% were females in controls). In the second RS_mult_ model, we omitted age and sex. BMI and ancestry were both significant and yielded a score with OR = 1.24 (95% CI [1.06, 1.46], *p* = 6.34 × 10^−3^) and AUC = 0.56. The RS_mult_ model focusing on clinical/disease variables (AUC = 0.8, OR = 2.85 (95% CI [2.46, 3.31], *p* = 8.76 × 10^−44^) performed better than the models focusing on ECG traits (AUC = 0.66, OR = 1.65 (95% CI [1.45, 1.88], *p* = 4 × 10^−14^), electrolytes (AUC = 0.64, OR = 1.76 (95% CI [1.52, 2.04], *p* = 1.9 × 10^−14^), sugars/lipids (AUC = 0.75, OR = 2.04 (95% CI [1.8, 2.32], *p* = 9.88 × 10^−29^). The model that included the blood and inflammatory variables did not show significant results. The global model showed a negligible improvement in performance over the model with clinical/disease variables (AUC = 0.81, OR = 2.85 (95% CI [2.46, 3.3], *p* = 2.2 × 10^−44^). The OR_decile_ was the highest for the global model (OR_decile =_ 9.57). Xgboost model included all variables. The global RS_xgboost_ outperformed the global RS_mult_ (AUC = 0.84, OR = 2.06 (95% CI [1.87, 2.28], *p* = 2.1 × 10^−46^). Most importantly, the OR_decile_ for xgboost was 13.99, which was substantially higher than the global RS_mult_ (i.e., 9.57). The effect sizes for the multivariate regression and variable importance for xgboost are shown in Supplementary Table S1. Finally, we performed a risk score analysis excluding AF patients from the dataset, but the obtained performance did not change (data not shown).

## 4. Discussion

**Figure 6:**
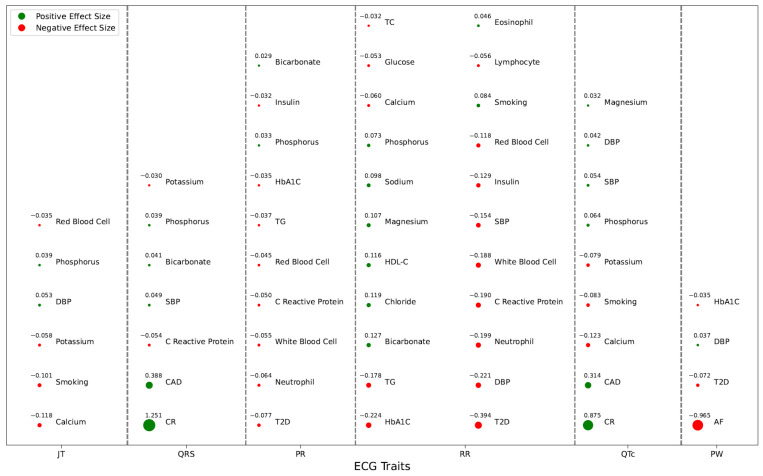
Summary of all Bonferroni-significant associations. Circle size is proportional to the effect size. The value of effect sizes is shown above the circles.

In this study, we performed association analysis between ECG traits (RR, PR, QRS, QTc, PW, and JT) and several clinical biomarkers and diseases. We used the QBB dataset of 13,827 participants with available ECG data. We tested three types of biomarkers: serum electrolytes (chloride, magnesium, potassium, sodium, calcium, phosphorus, and bicarbonate), sugars/lipids (glucose, HbA1C, insulin, LDL-C, HDL-C, total cholesterol, and TG), and blood and inflammatory (eosinophil, basophil, lymphocyte, monocyte, neutrophil, red blood cells, white blood cells, and C-reactive protein). The clinical and disease traits that were tested with ECG traits were AF, AR, CM, T2D, CAD, smoking, hyperthyroidism, stroke, SBP, and DBP. This is the first and largest such a study in the Middle East and North Africa region. Participants in the QBB cohort had Arab, African, and South Asian origins. The summary of significant associations and their directions with ECG traits is shown in Figure 6.

Serum electrolyte imbalances have been reported to be associated with ECG abnormalities and cardiac arrhythmias [22,23]. Mild hyperkalemia was associated with a narrow QTc interval [23]. This is concordant with what was observed in our study. QTc was inversely and significantly associated with potassium levels. However, potassium levels were not associated with PR, as previously discussed [13,23]. Consistent with the literature, lower calcium levels (hypocalcemia) were associated with prolongation of QTc [22,24]. Lower levels of calcium were also associated with elevated JT and RR. Heart rate, which is inversely proportional to RR, was previously shown to correlate with lower levels of calcium [25]. In our study, heart rate increase was associated with a rise in all serum electrolytes (except for calcium), with bicarbonate and chloride being the most statistically significant. Phosphorus rise was associated with prolonged QTc. In the Third National Health and Nutrition Survey (NHANES III) and the Atherosclerosis Risk in Communities (ARIC) study, phosphorus was positively associated with longer QTc, which is consistent with our data [24]. However, in patients undergoing hemodialysis, longer QTc was associated with lower phosphorus serum levels [26]. This suggests that the associations we identified may be valid for relatively healthy participants, and different relationships may be observed depending on the presence of certain diseases.

Sugars, lipids, and BP traits are known risk factors for CVD and T2D. CVD and T2D can lead to cardiac arrhythmias [27]. AF, which is the most common form of arrhythmias, is associated with a range of CVD [28]. In our study, all sugars/lipids/BP were associated with heart rate except LDL-C. An increase in these traits was associated with an increase in heart rate, except for HDL-C, which was negatively correlated with heart rate. In a recent study with a relatively small sample size, a positive correlation was observed between heart rate and HDL-C, LDL-C, BMI, and TG [29]. Our results should be more accurate because of our larger sample size (40× higher) and thus higher statistical power. The good cholesterol HDL-C is considered a protective factor against CVD, and it is expected that it has an impact on decreasing heart rate. QTc was only associated with BP traits where the correlation was positive (higher blood pressure associated with longer QTc). Elevations in SBP and DBP can disrupt ventricular repolarization, leading to the prolongation of the QT interval [30]. Prolonged PR intervals increase susceptibility to AF [31,32]. It was previously shown that enhanced PI3K activation reduced PR intervals in cross-bred transgenic mice [33]. Therefore, PI3K activation by insulin may avert AF and improve cardiac rhythm [31]. Our results add evidence to this hypothesis, where we showed that an increase in insulin and HbA1C levels was associated with reduced PR intervals.

PR interval was negatively associated with red blood cells, neutrophils, and C-reactive protein. The decrease in any of these biomarkers may lead to a prolonged PR. The decrease in RR due to an increase in red blood cells, neutrophils, and C-reactive protein resulted in only a PR interval decrease, while QRS, QTc, PW, and JT remained relatively unchanged. The other types of white blood cells were not associated with any ECG trait. The increase in red blood cells is expected to be associated with an increase in heart rate (decrease in RR). When tissues receive insufficient oxygen, the body may attempt to compensate by increasing the heart rate to pump more oxygenated blood to the tissues.

ECG alterations have been previously observed in T2D, CVD, CM, and other diseases [34]. For example, long QTc, QT dispersion, and left ventricular hypertrophy may be observed in T2D patients [35]. In our study, PR, RR, and PW intervals were the only ECG variables that decreased in T2D patients. In CAD patients, QRS and QTc were the only ECG traits that showed significant associations. An increase in QRS and QTc was observed in CAD patients. Like CAD, CM patients showed higher QRS and QTc levels. As expected, the increase in QRS and QTc was greater in CM patients than in CAD patients. AF patients, despite their small numbers, showed a significant decrease in PW intervals. Long QRS and QTc were found to be among the strongest predictors of CAD events in postmenopausal women [36] concordant with what was observed in our study, which generalizes this finding to the general population (not only postmenopausal women). These two variables were also identified as the dominant mortality predictors [36]. They can be used clinically to improve the prognosis of CAD patients.

The risk prediction of CAD using various well-established risk factors is important for early detection and prevention. One commonly used risk score is QRISK3 [19]. Additional risk factors can improve the performance of risk scores for CAD. ECG traits were previously used to predict the level of coronary artery calcium, and they provided good performance [16]. ECG abnormality risk scores were also shown to predict mortality risk in the elderly [37]. Recently, a deep learning model was developed using 12-lead ECGs and predicted 5-year atherosclerotic disease with an AUC of 0.67 [38]. Our results showed a good predictive power for CAD using ECG traits only (i.e., AUC = 0.66). OR for the top decile compared to the remaining deciles was 3.76 for the model that includes ECG traits only, which means a 3.76-fold risk increase in people with the highest risk score values. The performance of the risk scores developed using serum electrolytes, sugars/lipids, or clinical/disease traits all outperformed the ECG-based risk score. The global risk score, which showed the greatest predictive performance (AUC = 0.81 and OR_decile_ = 9.57), contained T2D, stroke, SBP, DBP, RR, PR, QRS, QTc, magnesium, potassium, smoking, and HbA1C. The machine-learning model, xgboost, outperformed the multivariate logistic regression (AUC = 0.84 and OR_decile_ = 13.99). The model included serum electrolytes, sugars/lipids, demographics, and clinical/disease risk factors. Both multivariate and xgboost models can be easily used in clinical settings. However, it is important to validate our developed risk scores in independent cohorts from the same region. Finally, the validation and utility of integrating ECG risk scores, genetic risk scores, and clinical scores remains to be seen in future longitudinal studies for CAD and other diseases.

This study has a few limitations. The sample size for diseases (AF, CM, AR, CAD, and stroke) is small. In the risk score analysis, ideally, the cases and controls should be age- and sex-matched, which was not the case in our study. Selecting a subset of controls to match our cases would have reduced the sample size drastically, especially for the disease categories. Since this is a retrospective study, the validation of the risk scores needs further investigation using longitudinal datasets.

## 5. Conclusions

Our study is the largest and the first study to investigate ECG trait associations with sugars, lipids, BP, blood and inflammatory biomarkers, CAD, T2D, and arrhythmias in a Middle Eastern cohort. Significant associations were identified with different ECG traits. RR was the ECG trait that showed significant associations with the highest number of variables. T2D, HbA1C, and triglycerides showed the largest negative effect size with RR. Importantly, QTc was shown to be longer in CAD patients but showed a negative correlation with calcium levels. Interestingly, the JT interval, part of the QTc interval, was negatively associated with calcium levels and smoking. Risk scores for CAD showed great predictive power. They included ECG traits, demographics, serum electrolytes, sugars, lipids, and clinical and disease traits (T2D, HbA1C, TC, QRS, SBP, QTc, smoking, glucose, RR, potassium, LDL-C, PW, TG, BMI, DBP, insulin, stroke, HDL-C, and PR). Implementation of these scores in clinical practice should help in setting tailored prevention and treatment plans for everyone.

## Figures and Tables

**Table 1:** Cohort characteristics and variable distributions.

	Mean ± SD/N (%)
Sex (male)	6340 (44.46)
Age	40.12 ± 13.11
BMI	29.6 ± 6.16
AF	67 (0.005)
AR	156 (0.011)
CR	48 (0.003)
CAD	527 (0.038)
T2D	3308 (0.236)
Hyperthyroidism	1369 (0.098)
Smoking	3615 (0.258)
Stroke	92 (0.007)
RR	905.29 ± 131.23
PR	162 ± 22.55
QRS	93.28 ± 10.63
QTc	402.29 ± 18.56
PW	116.15 ± 11.13
JT	309.01 ± 19.78

**Table 2:** Associations between ECG traits and demographic data.

	Sex		Age		BMI		Ancestry
	*b*	*p*	*b*	*p*	*b*	*p*	*p*
RR	−0.44	6.04 × 10^−150^	−0.06	3.81 × 10^−13^	−0.15	6.27 × 10^−66^	0.42
PR	−0.24	3.12 × 10^−43^	0.23	8.67 × 10^−163^	0.18	1.86 × 10^−96^	7.99 × 10^−19^
QRS	−0.71	0	0.12	1.20 × 10^−42^	0.10	1.07 × 10^−31^	3.52 × 10^−14^
QTc	0.56	1.29 × 10^−247^	0.26	3.43 × 10^−218^	0.20	7.58 × 10^−122^	2.12 × 10^−4^
PW	−0.29	2.05 × 10^−66^	0.23	2.01 × 10^−168^	0.27	9.14 × 10^−222^	2.55 × 10^−6^
JT	0.91	0	0.18	1.84 × 10^−106^	0.13	8.13 × 10^−55^	0.04

The sex variable was coded as 1 for females and 0 for males. Negative effect size means a decrease in ECG traits in females.

**Table 3:** Multivariate regression and machine-learning risk score models to predict CAD using several combinations of risk factors.

	Risk Score					
	OR	OR 95% CI	*p*	OR Decile	AUC	AUC 95% CI
Multivariate regression						
BMI + Ancestry	1.24	[1.06, 1.46]	6.34 × 10^−3^	1.36	0.56	[0.52, 0.61]
Sex + Age	3.84	[3.21, 4.59]	3.55 × 10^−49^	11.73	0.84	[0.81, 0.87]
PR + QRS + QTc	1.65	[1.45, 1.88]	4.00 × 10^−14^	3.76	0.66	[0.61, 0.7]
HDL + HbA1C	2.04	[1.8, 2.32]	9.88 × 10^−29^	7.57	0.75	[0.7, 0.79]
Chloride + Magnesium + Potassium + Calcium	1.76	[1.52, 2.04]	1.94 × 10^−14^	4.32	0.64	[0.59, 0.69]
SBP + DBP + Smoking + T2D + Stroke	2.85	[2.46, 3.31]	8.76 × 10^−44^	9.29	0.8	[0.77, 0.84]
SBP + DBP + Smoking + T2D + Stroke + RR + PR + QRS + QTc + HbA1C + Magnesium + Potassium + Ancestry	2.85	[2.46, 3.3]	2.20 × 10^−44^	9.57	0.81	[0.77, 0.85]
xgboost						
T2D + HbA1C + TC + QRS + SBP + QTc + Smoking + Glucose + RR + Potassium + LDL-C + PW + TG + BMI + DBP + Insulin + Stroke + HDL-C + PR	2.06	[1.87, 2.28]	2.10 × 10^−46^	13.99	0.84	[0.81, 0.88]

TC: total cholesterol; TG: triglyceride; SBP: systolic blood pressure; DBP: diastolic blood pressure.

## Data Availability

The data are not available in public repositories. They can be accessed through application to the Qatar Biobank through an established ISO-certified process by submitting a request online, subject to institutional review board approval by the Qatar Biobank. To submit a request, see https://www.qatarbiobank.org.qa/research/how-to-apply-new/ (accessed on 28 December 2023).

## Supplementary Materials

The following supporting information can be downloaded at https://www.mdpi.com/article/10.3390/jcm13010276/s1, Table S1: Effect sizes and variable importance of the traits included in the risk scores using multivariate regression and xgboost.
