# Supplementary material for: Correlations between Resting Electrocardiogram Findings and Disease Profiles: Insights from the Qatar Biobank Cohort
Source: J Clin Med. 2024 Jan 3;13(1):276. doi: 10.3390/jcm13010276 (PMC10779868; doi:10.3390/jcm13010276)
Supplement: Supplementary file 1 [file jcm-13-00276-s001.zip › jcm-2767812-SI.pdf]

Table S1. Effect sizes and variable importance of the traits included in the risk scores using multivariate regression and xgboost.

Multivariate Regression			xgboost			
**Trait**	**Effect size**	**P-value**	**Trait**	**Gain**	**Cover**	**Frequency**
T2D	0.995	3.20E-10	T2D	0.2602	2.63E-01	0.15
Smoking	0.760	1.66E-08	HbA1C	0.2210	2.11E-01	0.13
QTc	0.018	5.95E-08	TC	0.1147	5.52E-02	0.11
SBP	0.020	5.25E-07	QRS	0.0922	1.92E-01	0.18
HbA1C	0.220	8.98E-07	SBP	0.0917	6.44E-02	0.08
QRS	0.018	4.02E-05	QTc	0.0677	6.94E-02	0.10
Potassium	0.647	6.35E-05	Smoking	0.0365	2.11E-02	0.05
DBP	-0.018	5.01E-03	Fasting Glucose	0.0267	4.68E-02	0.05
RR	0.001	8.01E-03	RR	0.0261	8.75E-03	0.04
Stroke	0.833	1.39E-02	Potassium	0.0226	2.32E-02	0.04
SAS	0.396	2.28E-02	LDL-C	0.0206	7.94E-03	0.02
PR	0.006	2.37E-02	PW	0.0079	4.97E-03	0.01
Magnesium	1.880	2.62E-02	TG	0.0044	4.84E-04	0.01
			BMI	0.0018	2.94E-02	0.01
			DBP	0.0017	6.86E-04	0.00
			Insulin	0.0016	1.82E-04	0.00
			Stroke	0.0014	6.08E-04	0.00
			HDL-C	0.0007	4.22E-04	0.00
			PR	0.0006	2.98E-05	0.00
